# Conversion of failed hemiarthroplasty to total hip arthroplasty: A short to mid-term follow-up study

**DOI:** 10.4103/0019-5413.41852

**Published:** 2008

**Authors:** Amite Pankaj, Rajesh Malhotra, Surya Bhan

**Affiliations:** Department of Orthopedics, GTB Hospital and University College of Medical Sciences, Delhi - 110095, and Unversity of Delhi; 1 Department of Orthopedics, All India Institute of Medical Sciences, Ansari Nagar, New Delhi - 110 029, India

**Keywords:** Conversion, hemiarthroplasty, total hip arthroplasty

## Abstract

**Background:**

The conversion of hemiarthroplasty (unipolar or bipolar) of the hip to total hip replacement has been reported to be associated with very high rates of intra- and postoperative complications. We present a prospective analysis of the outcome of conversion surgery in patients with failed hemiarthroplasty.

**Materials and Methods:**

Forty-four cases, 30 women and 14 men, average age 62 years (range 42-75 years) of failed hemiarthroplasty were converted to total hip replacement between January 1998 and December 2004. Groin pain was the main presenting complaint in the majority of the patients (24 out of 44). Six patients had infection and were operated with staged procedure. All acetabular and the majority (86.5%) of femoral components used in our series were uncemented.

**Results:**

After an average follow-up of 6.4 years (range, two to nine years) Harris hip scores improved from 38 (range 15-62) preoperatively to 86 (range 38 to 100) and 22 (50%) patients were community ambulators without support while 17 (38%) needed minimal support of cane. Fifteen out of 18 (83%) patients who had isolated groin pain preoperatively experienced no pain postoperatively while three patients (17%) reported only partial improvement. Intraoperative and postoperative complications included iatrogenic fracture of the femur in two, femoral perforation in two, partial trochanteric avulsion in two, fracture of the acetabular floor in three hips, and postoperative dislocation in one. None of these complications resulted in a poor long-term outcome. The rate of loosening in our series was 2.3% (one out of 44) after a mean follow-up of 6.4 years with a mean survival of 97.4% at 72 months.

**Conclusion:**

Conversion of symptomatic hemiarthroplasty to total hip arthroplasty is a safe option that gives good functional results, with marginally higher rates of intra-operative complications. The patients should be warned of the possibility of incomplete relief of groin pain postoperatively.

## INTRODUCTION

Hemiarthroplasty (unipolar or bipolar) of the hip is a commonly performed procedure in elderly patients with intracapsular displaced fractures of the neck of the femur with good short-term results with regard to pain relief, return to activity, morbidity and mortality.^1^–^3^ Although bipolar hemiarthroplasty has been advocated by some for treatment of various arthritic conditions of the hip joint the results have not been very gratifying and it has largely been given up in favor of total hip arthroplasty (THA).^4^ Long-term problems associated with hemiarthroplasty include progressive acetabular cartilage degeneration and concomitant groin pain, protrusio, stem loosening and subsidence; and very poor results have been reported in active patients.

Bipolar arthroplasty was considered to improve the long-term outcome of hemiarthroplasty as a result of less wear of the metal-cartilage interface by providing another interface (metal-polyethylene) inside the bipolar head. However, recent studies comparing bipolar to unipolar hemiarthroplasty show little difference between the two with regard to morbidity, mortality, or functional outcome.^5^ Current evidence is emerging that THA may be a better choice for patients of intracapsular fractures of the neck of the femur in elderly age group (60-75 years) who are mentally competent, relatively healthy, active, capable of living independently and have a long life expectancy.^6^,^7^

The indications for conversion of hemiarthroplasty to THA include acetabular erosions and protrusio causing groin pain, femoral loosening and subsidence causing thigh pain and the typical “start-up” pain, dislocation, breakage of implant leading to loss of function, peri-prosthetic fracture and infection. Conversion of hemiarthroplasty is associated with high complication rates and loosening rates as against primary total hip arthroplasty.^8^–^11^ The purpose of the present study was to evaluate the functional outcome, survivorship at short to mid-term follow-up, and complication rates of conversion of hemiarthoplasty to THA in a tertiary care referral hospital.

## MATERIALS AND METHODS

Fifty-four consecutive patients of failed hemiarthroplasty of the hip were enrolled for the study between January 1998 and December 2004. Ten cases that did not have a minimum follow-up of two years either as a result of death or attrition were excluded leaving 44 patients for further evaluation. The average age at the time of conversion surgery was 62 years (range 42-75 years). There were 30 women and 14 men in the study group.

All patients being planned for a conversion surgery were investigated according to a standard protocol. A detailed history was noted including the indication for the primary surgery and duration of symptoms. The indication for primary surgery was a displaced femoral neck fracture in 40 and arthritides in four patients. The average time since primary surgery was 84 months (range 14 months to 12 years). Eighteen patients (41%) had groin pain as the presenting symptom, 11 (25%) had thigh pain, six (14%) had both groin and thigh pain and nine patients (20%) presented with loss of function. The average preoperative Harris Hip Score was 38 (range 15-62). The average preoperative shortening was 2.4 cm (range 0-4 cm). The majority of the patients (24 out of 44), were in ASA Grade I, 12 in ASA Grade 2 and eight were in ASA Grade 3. Twenty-four patients were community ambulators (with support in all but one), 11 were homebound and nine were bedridden. Anteroposterior radiographs of the pelvis, anteroposterior and lateral radiographs of the involved hip with thigh were obtained. Routine hemogram including ESR and CRP were also done. The erythrocyte sedimentation rate (ESR) and C-reative protein (CRP) were elevated in eight and seven patients respectively. In case of elevated levels of ESR and CRP, the tests were repeated after three weeks and if elevated, a presumptive diagnosis of septic loosening was made. Diagnostic aspiration from the hip was not done in any patient. All patients were admitted two days prior to surgery after getting the preanesthetic clearance. Preoperative decision regarding the choice of implant was made depending upon the presence/absence of calcar, extent of osteolysis of proximal femur, cortical perforations, if any, and the degree of acetabular erosions. Primary total hip replacement prostheses were used only when the proximal femur was near normal in terms of the above mentioned features (16 hips). In patients with calcar resorption a calcar-replacing revision prosthesis was chosen. In patients having osteolysis of proximal femur a revision prosthesis was selected while a primary prosthesis was selected if there was no osteolysis. In patients with cortical erosion/perforations at the tip of the prosthesis a revision femoral stem was so selected that it bypassed the defect by at least 5 centimeters. Acetabular reconstruction devices such as antiprotrusio rings/cages were not required and primary cementless acetabular component was used in all hips. The distribution of prostheses that were used is given in Table 1.

**Table 1:** Distribution of Prostheses used (*n* = 44)

Primary total hip replacement implant	16 (36.5%)
Cemented	None
Cementless (Versys and Trilogy)	10
Hybrid (CPT stem and Trilogy)	6
Revision total hip replacement implant	28 (63.5%)
HA coated Interlocking stem (IOTA stem and Trilogy cup)	18
Fully porous coated stem (Versys stem and Trilogy cup)	8
S-ROM	2

**Figure 1:**
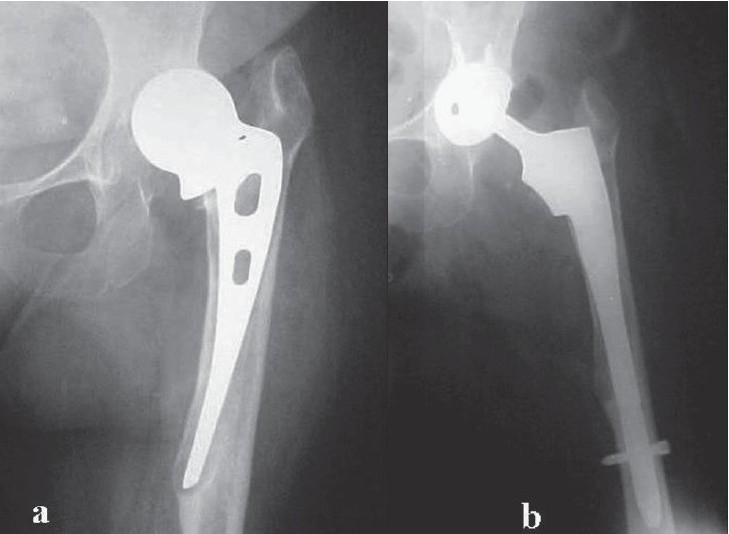
(a) A case of failed Austin Moore prosthesis with subsidence calcar resorption and cortical thinning at the tip, (b) Calcar-replacing revision hip prosthesis with interlocking stem was performed. Note that the tip of revision stem is at a distance well beyond the tip of the failed endoprosthesis

All procedures were performed through posterior approach using a standard length incision with the patient in lateral position and every effort was made to utilize the previous incision whenever possible. All procedures were done under combined spinal epidural anesthesia. All procedures were performed according to a standard technique with minor variations as and when required. The skin and subcutaneous tissues were incised in one line and the plane was established between the subcutaneous tissues and gluteus fascia for 1-2 cm on both sides. The gluteus maximus fibers were split and tensor fascia lata was incised. The sciatic nerve was identified and protected. In cases previously operated through the posterior approach no distinction could be made of the external rotators and the capsule and the scar tissue was elevated ‘en masse’ from the intertrochanteric ridge posteriorly, thus exposing the hip joint and avoiding the inadvertent damage to the sciatic nerve. Any collection/fluid was collected in a syringe and was sent for culture and gram stain. Gluteus maximus insertion was partially released for better exposure and restoration of limb length. Next, the scar tissue was released meticulously from all around the prosthesis. The prosthetic joint was dislocated posteriorly by flexing, adducting and internally rotating the hip. Anterior capsule and scar tissue was removed with diathermy at this stage. The prosthesis, which was found to be loose in a vast majority, was removed and the samples from the intramedullary canal were sent for histoplathological and microbiological work-up. Fibrous membrane was removed with long curettes. Austin Moore prostheses were removed in 32, non-modular cemented bipolar in eight, and modular cemented bipolar in four hips. The distal canal was opened with a hand reamer/drill thus perforating the sclerotic bone at the tip of the loose prosthesis and utmost care was taken to avoid the tendency to go through the path of least resistance, which may have caused a cortical perforation [Figure 1a]. The canal was then prepared according to the hip system being used in a particular patient. While using a revision hip system, length of the prosthesis was so chosen as to bypass the tip of the previous stem by 5 cm [Figure 1b]. The acetabular preparation was done in usual fashion. Under-reaming by 2 mm was done while using cementless implant and two screws were used as supplemental fixation. Primary hip prostheses were used in 16 and revision implants in the remaining 28 patients.

**Figure 2:**
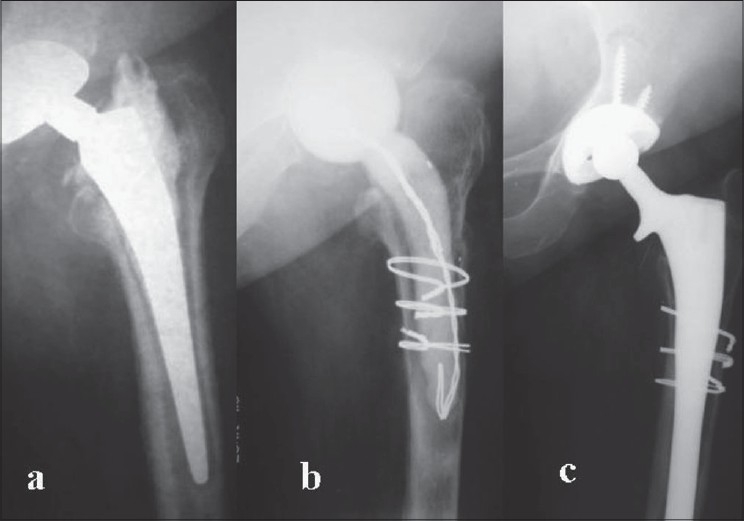
Two-stage revision of infected bipolar arthroplasty. (a) Preoperative radiograph showing septic loosening of the cemented bipolar prosthesis, (b) First stage of revision surgery with insertion of antibiotic-impregnated cement spacer; fracture of the proximal femur was stabilized with cerclage wiring, (c) Second stage revision surgery with a long hydroxyapatite coated femoral stem

Additional procedures that were done included release of iliopsoas in 24 patients; extended trochanteric osteotomy in five patients to facilitate removal of the femoral implants (broken implant in four and a well-fixed intact prosthesis in one), and cerclage wiring of the femur in two hips to treat iatrogenic fractures of the femur [Figure 2] that occurred while dislocating the prostheses. Release of iliopsoas (partial or complete) was done to (a) improve exposure and, (b) to correct limb length discrepancy; decision for iliopsoas release was taken intraoperatively. Partial trochanteric avulsion was observed in two patients and was treated with fixation with cerclage wiring. In one patient it occurred while attempting to reduce the trial components and the reduction was very tight and in another it occurred during femoral preparation.

Based on the preoperative work-up, seven hips were presumed to be potentially infected and a two-stage revision approach was adopted. After exposing the joint the tissue was sent for frozen section and aspirated fluid was subjected to cytological examination as well as gram staining. In case the gram stain was found to be positive (one hip) or the frozen section and aspirated fluid showed presence of neutrophilic response (one or more neutrophil polymorphs per high-power field, as seen in five hips including the one with a positive gram stain), we adopted a two-stage revision approach. Out of seven patients who had a presumptive diagnosis of infection on the basis of preoperative investigations, only one patient was found to be suitable for implantation and the rest were treated with a thorough debridement, cleaning of intra-medullary canal, and insertion of an antibiotic- (Vancomycin 2g in 40g of CMW 1 cement) impregnated cement spacer prepared in the operation theatre [Figure 2b]. The patients were started on intravenous (iv) antibiotics (cefazolin and amikacin) empirically and were switched over to definitive antibiotics based on the culture sensitivity reports. Only three of the six hips grew bacteria [(methicillin sensitive staphylococcus aureus (MSSA) in two and Klebsiella in one)]. Intravenous antibiotics were continued for two weeks and followed by oral antibiotics for another four weeks. ESR and C-reactive protein levels were obtained after six weeks and if with in normal range, second stage of revision was planned. Second stage was performed at an interval varying from six to 10 weeks. The same guidelines were followed during the second-stage procedure and if there was any suspicion regarding the infection, the spacer was exchanged for another spacer (n = 1).

Preoperative prophylaxis against infection was given to all patients (Ceftriaxone 1g intravenously before the surgery followed by 1g twice daily for three days). Subcutaneous enoxaparin (40 mg once daily) starting on the day of surgery was given to all patients for seven to 10 days in addition to antiembolism stockings as prophylaxis against deep vein thrombosis (DVT). Oral aspirin (150 mg once daily) was given for three to four weeks after discontinuation of enoxaparin. No routine screening for DVT was done; however, Doppler ultrasonography was carried out in the event of clinical suspicion of DVT. Early mobilization was used both to prevent DVT and to hasten the functional recovery (in-bed mobilization starting from the second postoperative day in all patients except when specifically contraindicated). Full weight-bearing was allowed from the third day onwards in all cases with hybrid arthroplasty (*n* = 6). Patients with cementless components (*n* = 38) were allowed full weight-bearing after 12 weeks.

Complications local to each joint including fracture, dislocation, superficial wound infection, deep wound infection around the prosthesis and incidence of heterotopic ossification (HO) were recorded. We did not use prophylaxis for heterotopic ossification (HO). Systemic complications including cardiac, gastrointestinal complications, cerebrovascular accidents, phlebitis/pulmonary embolism, and urinary tract infection were also noted.

### Radiographic Evaluation

One of the investigators evaluated the postoperative radiographs obtained at one month postoperatively. The parameters recorded were cup abduction angle and alignment of the stem. Stem alignment was measured as the angle between the femoral stem and the long axis of the femur on anteroposterior radiographs and was classified as varus, neutral or valgus. On the lateral radiograph, the stem alignment was classified as anterior, neutral or posterior. Limb-length discrepancy was also recorded by measuring the distance of the upper margin of the lesser trochanter from the inter-teardrop line. Follow-up radiographs were evaluated for stem subsidence, appearance/progression of radiolucent lines, osteolysis, stress-shielding of proximal femur, loosening and bony ingrowth according to the criteria described by Engh *et al*.^12^

### Postoperative follow-up

The patients had clinical and radiographic evaluations at one, three, six months, one year, and annually thereafter following the conversion procedure and the second procedure in the two-stage group. Since the patients who received a cementless THA were allowed to bear full weight only after three months from surgery, the Harris Hip Score (HHS) was recorded at six months postoperatively in all patients. Although the HHS at final follow up of 2 year was recorded for all patients, the six-month HHS was used to evaluate the results of conversion procedure in terms of relief of groin pain and functional improvement. The use of walking aid was also recorded. Incidence of heterotopic ossification at any point during follow-up was recorded and classified as described by Brooker.^13^ Any other complications and the details of any revision procedure were also noted.

## RESULTS

The indication for surgery in the majority of the patients was groin pain (18 patients); thigh pain was present in 11, six patients had both groin and thigh pain, and nine patients had loss of function and were unable to walk. A preoperative diagnosis of acetabular erosion and protrusio was made in 14 hips (32%), aseptic femoral loosening in 15 (34%), septic loosening in six (12%), prosthesis breakage in four (9%), dislocation in three (7%), and periprosthetic fracture in two hips (5%) [Table 2]. Preoperative HHS was 38 (range 15-62). An average shortening of 2.4 cm (range 0-4cm) was recorded preoperatively. Eight patients had an elevated ESR, CRP was found to be elevated in seven patients. Majority of the patients, 24 out of 44, were community ambulators (with support in all but one) but needed regular analgesics; 11 patients were homebound, and nine were bedridden.

**Table 2:** Etiology of hemiarthroplasty failure (*n* = 44)

Acetabular erosion and protrusio	14 (32%)
Aseptic femoral loosening	15 (34%)
Infection	6 (12%)
Dislocation	3 (7%)
Prosthesis breakage	4 (9%)
Periprosthetic fracture	2 (5%)

**Figure 3:**
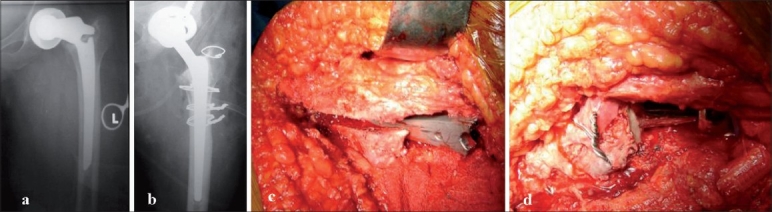
Conversion of a broken bipolar prosthesis, (a) pre- and (b) postoperative radiographs; extended trochanteric osteotomy was needed to extract the broken implant which was fixed with cerclage wiring, (c) intra-operative view of the extended trochanteric osteotomy, and (d) after fixation and completion of procedure

Out of seven patients with presumptive diagnosis of infection one patient was suitable for same stage implantation, five patients were treated with a two-stage revision and one patient needed a three-stage revision. Additional procedures that were done included release of iliopsoas in 24 patients; extended trochanteric osteotomy in five patients to facilitate removal of the femoral implants (broken implant in four and a well-fixed intact prosthesis in one) [Figure 3].

**Figure 4:**
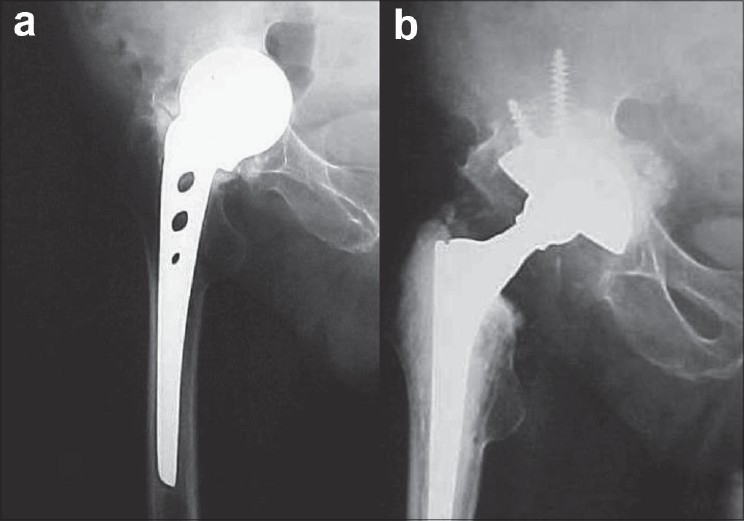
(a) Preoperative radiograph of a failed hemiarthroplasty with acetabular erosion and protrusio, (b) Two-year follow-up X-ray showing incorporation of morsellized allograft

Acetabular deficiency was seen in eight hips (Type II, American Academy of Orthopaedic Surgeons (AAOS) classification)^14^ and was managed with morsellized femoral head allograft [Figure 4]. Five femora had proximal calcar deficiency and were managed with a calcar-replacing prosthesis [Figure 1].

Intraoperative complications included iatrogenic fracture of the femur in two, femoral perforation in two, partial trochanteric avulsion in two and fracture of the acetabular floor in three hips. Fractures of the femur and trochanteric avulsion were treated by cerclage wiring and weight-bearing was delayed for three months in these patients. Fractures of the acetabular floor were also managed by delayed weight-bearing. All fractures healed without complications.

### Early postoperative complications

Superficial infection occurred in four hips and all cases responded to local wound care and antibiotics. Debridement with retention of prosthesis along with prolonged (three weeks) intravenous antibiotic therapy was required in three hips in view of persistent wound discharge. None of these patients had preoperative suspicion of infection. Dislocation occurred in one hip in the two-stage group on the third postoperative day of the second surgery, which was reduced by closed manipulation under anesthesia. The patient was mobilized with an abduction brace for three months and there was no recurrence thereafter.

Systemic complications included myocardial infarction in one, chest infection in one, urinary tract infection in two and clinically detectable DVT in two patients. No death was encountered in our study group that could be directly attributed to the surgery or its complications.

### Clinical follow-up

The mean follow-up was 6.4 years (range, 2-9 years). Harris hip scores improved from 38 (range 15-62) preoperatively to 92 (range 42 to 100) assessed six months postoperatively. Average HHS at the final follow-up (mean 6.4 years) was 86 (range 38 to 100). Fifteen out of 18 (83%) patients who had isolated groin pain preoperatively experienced no pain postoperatively while three patients (17%) reported only partial improvement. None of the patients had thigh pain postoperatively. At the last follow-up 22 (50%) patients were community ambulators without support, 17 (38%) patients could walk more than five blocks using cane, and five (12%) needed walker for ambulation and were homebound. Limb length discrepancy was corrected to within 1 cm in all but three patients. One patient needed a revision procedure (femoral revision) as a result of aseptic femoral loosening. The survivorship analysis with revision of either component as an end point demonstrated a mean survival of 97.4% at 72 months.

### Radiographic analysis

**Figure 5:**
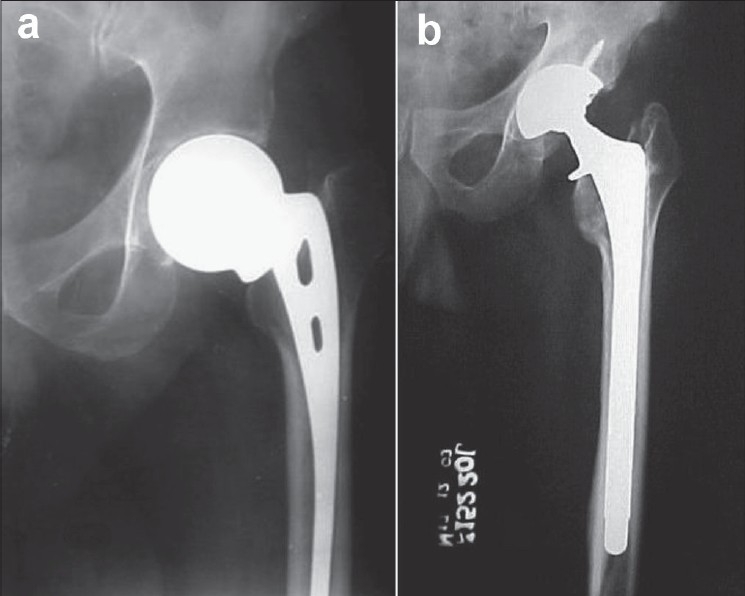
(a) Preoperative and, (b) 48 months follow-up X-rays of conversion of a loose Austin Moore prosthesis to a cementless total hip arthroplasty (THA) with long femoral stem. A non-progressive radiolucent line less than 2 mm in thickness was noted in Zone 7

Mean acetabular cup abduction angle was 44.36 ± 2.98 degrees (range 40-58 degrees). Alignment of femoral stems in both anteroposterior and lateral radiographs was neutral in 38, varus in four and valgus in two hips. The mean stem alignment angle was 0.52 ± 0.24 degrees of varus. Non-progressive radiolucent lines less than 2 mm were noted in Zone 7 in two cementless revision femoral stems [Figure 5] at final follow-up. None but one of the stems had subsidence of more than 0.5 cm. Incorporation of morsellized allograft was noticed in all eight acetabular reconstructions at the end of one year. Extended trochanteric osteotomy united in all five patients by three months. The incidence of HO was 15% (seven out of 44 hips), but no hip had HO greater than Brooker's Grade 2.

### DISCUSSION

Hemiarthroplasty (unipolar and bipolar) of the hip is a commonly performed procedure for the treatment of displaced intraarticular fractures of the neck of the femur in the elderly. The goal of treatment of displaced fractures of the neck of the femur is to return the patients to their pre-injury mobility status as early as possible and to minimize the risk of further operation.^15^ Austin Moore and Thompson prostheses have fulfilled these criteria for decades but have been associated with a poor quality of life in the long term with a very high incidence of groin and thigh pain in physically active elderly patients, largely a consequence of acetabular cartilage degeneration and stem loosening respectively.^6^,^16^,^17^ In our study group of failed hemiarthroplasty 41% patients complained of groin pain, 25% of thigh pain and 14% had both. Bipolar arthroplasty was introduced to improve the long-term outcome of hemiarthroplasty as a result of less wear of the metal-cartilage interface by providing another interface (metal-polyethylene) inside the bipolar head. However, recent studies comparing bipolar to unipolar hemiarthroplasty show little difference between the two with regard to morbidity, mortality, or functional outcome.^5^

Pain following hemiarthroplasty is usually due to one of two pathological processes: articular cartilage degeneration in the acetabulum or loosening of the prosthesis. These pathological processes are exacerbated by many factors including incongruence between the femoral head and the acetabulum, excessive neck length, impaction at the time of injury, cementation of the prosthesis, physiologically young active patients and shear forces between the prosthesis and the cartilage.^16^,^18^,^19^ In view of these observations current evidence is emerging that favors THA over hemiarthroplasty for treatment of displaced fractures of the neck of the femur in patients who are elderly but have an active physical life.^6^,^7^ The treatment of symptomatic hemiarthroplasty involves removal of the prosthesis and conversion to a total hip replacement and Cossey and Goodwin noted that conversion to THA would give satisfactory results.^20^ Other investigators, however, have reported that conversion of hemiarthroplasty to THA is associated with high complication and loosening rates as against primary total hip arthroplasty.^8^–^11^,^21^

Groin pain, which has been cited as the most common reason for conversion, does not seem to be relieved completely in every patient after conversion to THA. Sharkey *et al.*, while reporting the results of conversion of hemiarthroplasty to THA in 45 patients observed that 20% of the patients continued to have groin or buttock pain after THA and they could not identify a factor that would predict an unsuccessful result.^11^ In our study group 15 out of 18 (83%) patients who had isolated groin pain preoperatively experienced no pain postoperatively while three patients (17%) reported only partial improvement. Thigh pain, however, was relieved in all patients. Six patients who had both groin and thigh pain were relieved of both postoperatively. Sharkey *et al.*, suggested that patients should be warned of this contingency before the surgery, that they could experience some groin pain postoperatively.

One of the earliest studies on conversion arthroplasty, by Amstutz and Smith, noted very high incidence of intra as well as postoperative complications.^21^ They reported results of 41 patients with conversion arthroplasty. They had five intraoperative proximal femoral fractures, two perforations of the medial cortex with stem protrusion, two cases with instability, two cases with infection, three patients with deep venous thrombosis and six patients (14.6%) with progressive loosening. Three patients had required revision by the end of follow-up (mean of 36 months). Sierra and Cabanela^8^ in a larger series of 132 hemiarthroplasties that were converted to THA reported a 10% rate of loosening after a mean follow-up of 7.1 years and major complications in 45%, including 12 intaroperative femoral fractures (9%) and 13 dislocations (9.8%). They concluded that conversion of endoprostheses to THAs after femoral neck fractures is fraught with high complication and loosening rates and careful selection of patients for each type of arthroplasty (hemiarthroplasty versus THA) may help ameliorate the outcome of arthroplasty for this group of patients. We also observed a high rate of intra-operative complications with iatrogenic fracture of the femur in two, femoral perforation in two, partial trochanteric avulsion in two and fracture of the acetabular floor in three hips although none of these complications resulted in a poor long-term outcome. The rate of loosening in our series was 2.3% (one out of 44) after a mean follow-up of 6.4 years. Hammad and Abdel-Aal reported no loosening in their series of conversion arthroplasty in 47 patients after an average follow-up of 44 months.^9^ The reason for lower loosening rate in their series as against earlier studies^8^,^21^ as stated by them may have been as a result of better cementing technique and stem design. In addition, failure on the femoral side may be due to extensive resorption of the endosteal bone while the stem of the hemiarthroplasty was loose, or due to damage of the endosteal bone during revision.^22^

Furthermore, toggling of the stem may produce a thick fibrous membrane that is adherent and might not be completely removed at revision, with its remnants compromising the subsequent cemented fixation. Also, it had been suggested that fragments of such a fibrous membrane are metabolically very active, producing Prostaglandin E2, collagenase and Interleukin1b, all of which may contribute to resorption of adjacent bone.^23^,^24^ Our series differs from these studies in one respect i.e. all acetabular components and the majority (86.5%) of femoral components used in our series were uncemented and this was probably the reason for lower loosening rates.

The incidence of dislocation after conversion arthroplasty has been reported as varying from 0 to 50% in different series.^9^^,25^ We had one dislocation in the early postoperative period and we believe that occurrence of postoperative instability is technique-related.

Conversion of painful hemiarthroplasty gives good results with regard to the pain relief and functional scores.^9^ Our functional results were very encouraging with an average HHS of 86 at final follow-up and 22 (50%) patients were community ambulators without support while 17 (38%) patients could walk more than five blocks using minimal support.

We conclude that conversion of symptomatic hemiarthroplasty to THA is a safe option that gives good functional results, with marginally higher rates of intra-operative complications; and patients should be warned of the possibility of incomplete relief of groin pain postoperatively.
